# Supplementary material for: Circadian rhythms modulate the effect of eccentric exercise on rat soleus muscles
Source: PLoS One. 2022 Feb 25;17(2):e0264171. doi: 10.1371/journal.pone.0264171 (PMC8880858; doi:10.1371/journal.pone.0264171)
Supplement: S1 Raw images — (PDF) [file pone.0264171.s001.pdf]

S1 Raw images

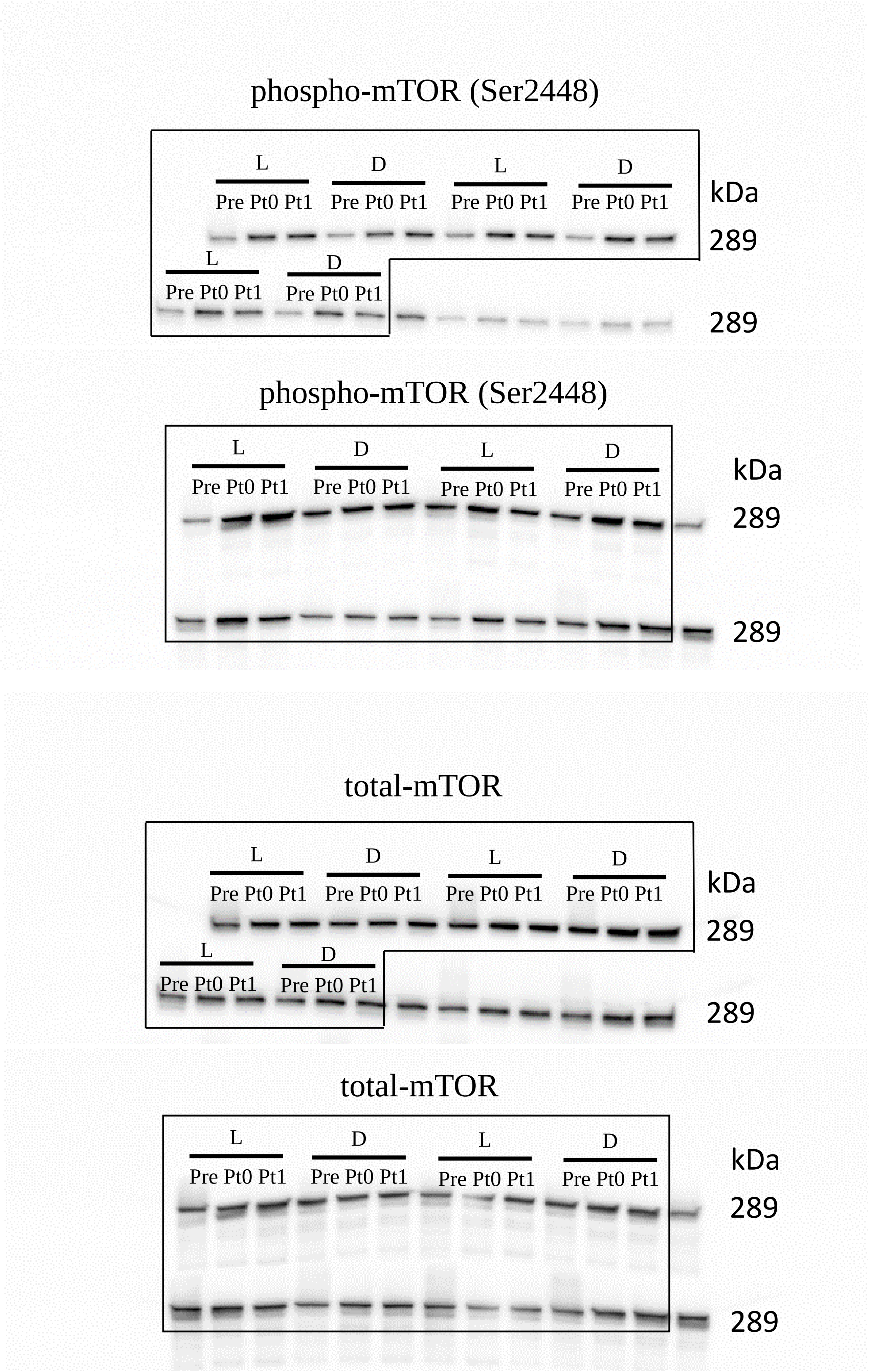

Fig 3a

S1 Raw images

phospho-p70S6K (Thr389)

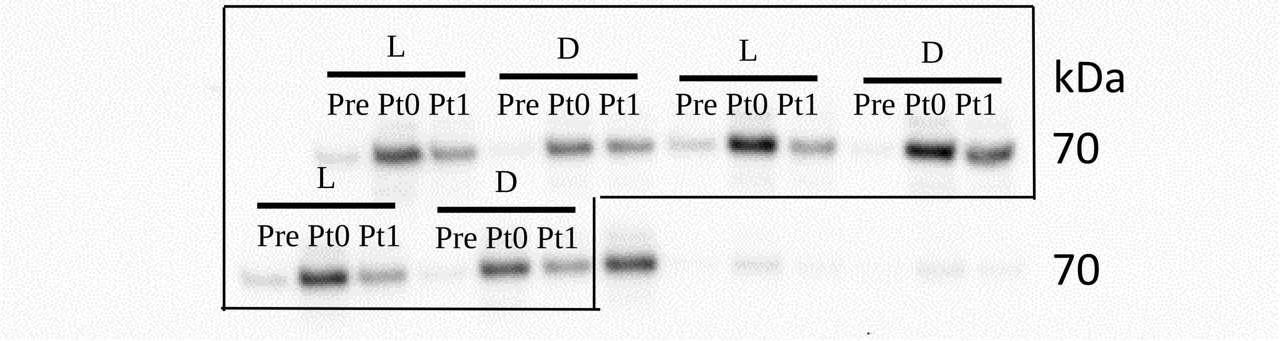

phospho-p70S6K (Thr389)

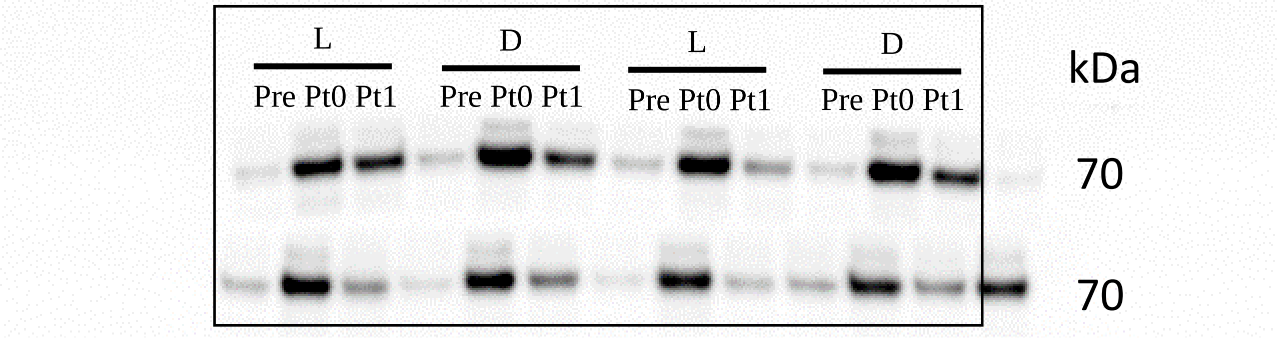

total-p70S6K

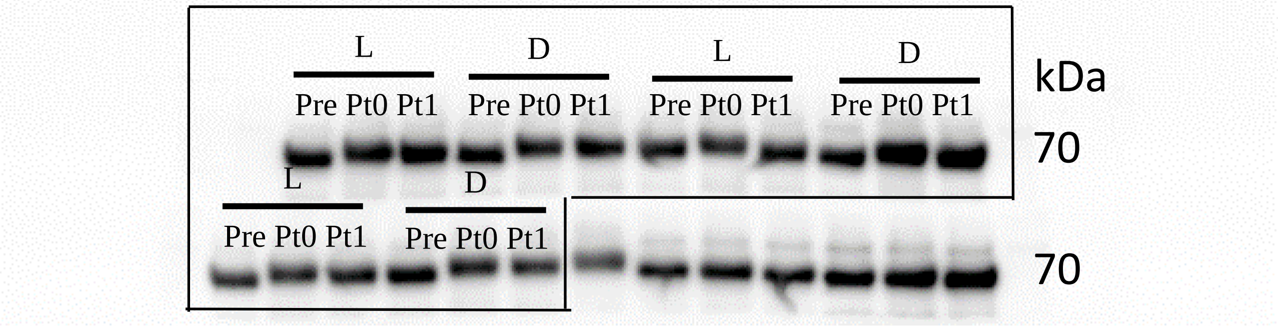

total-p70S6K

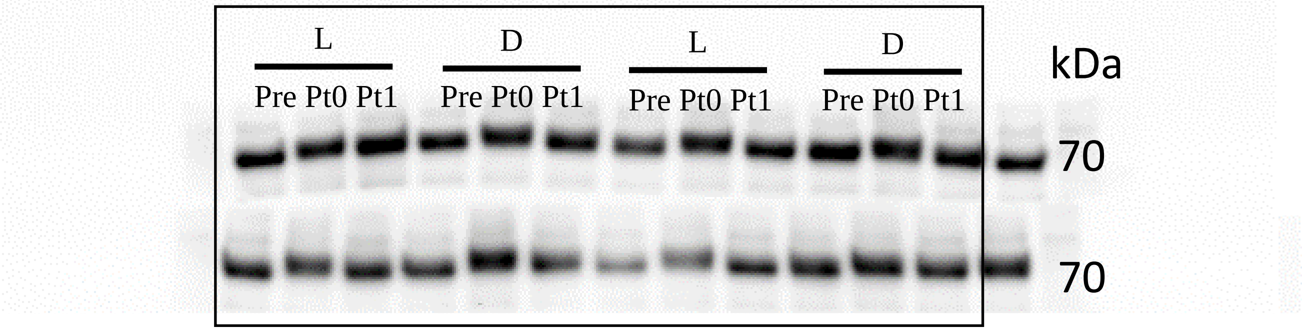

Fig 3b

phospho-4EBP1 (T37/46)

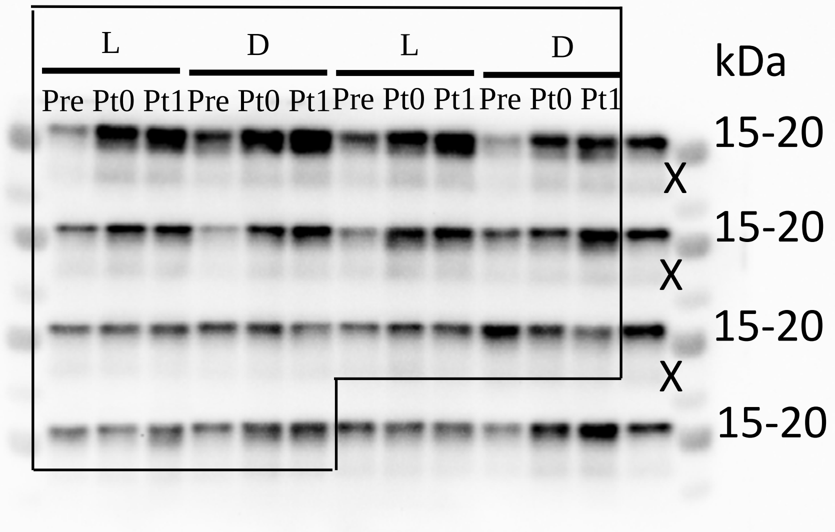

total-4EBP1

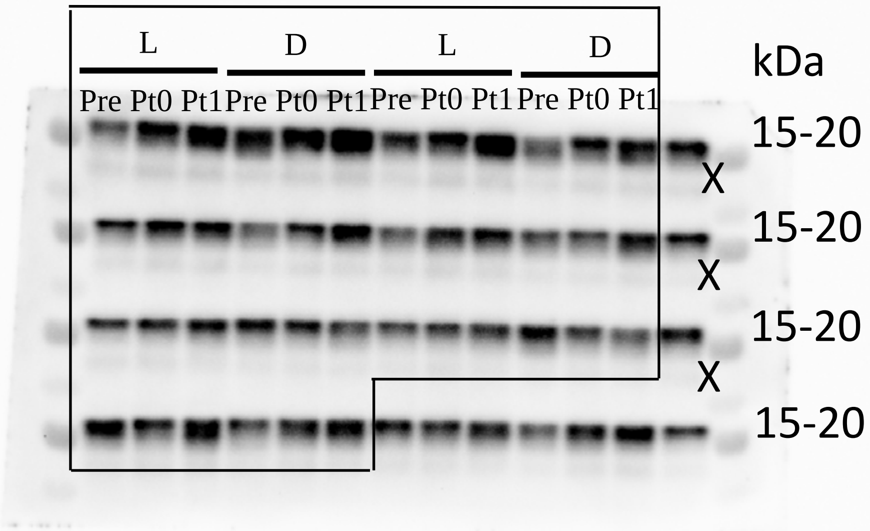

Fig 3c

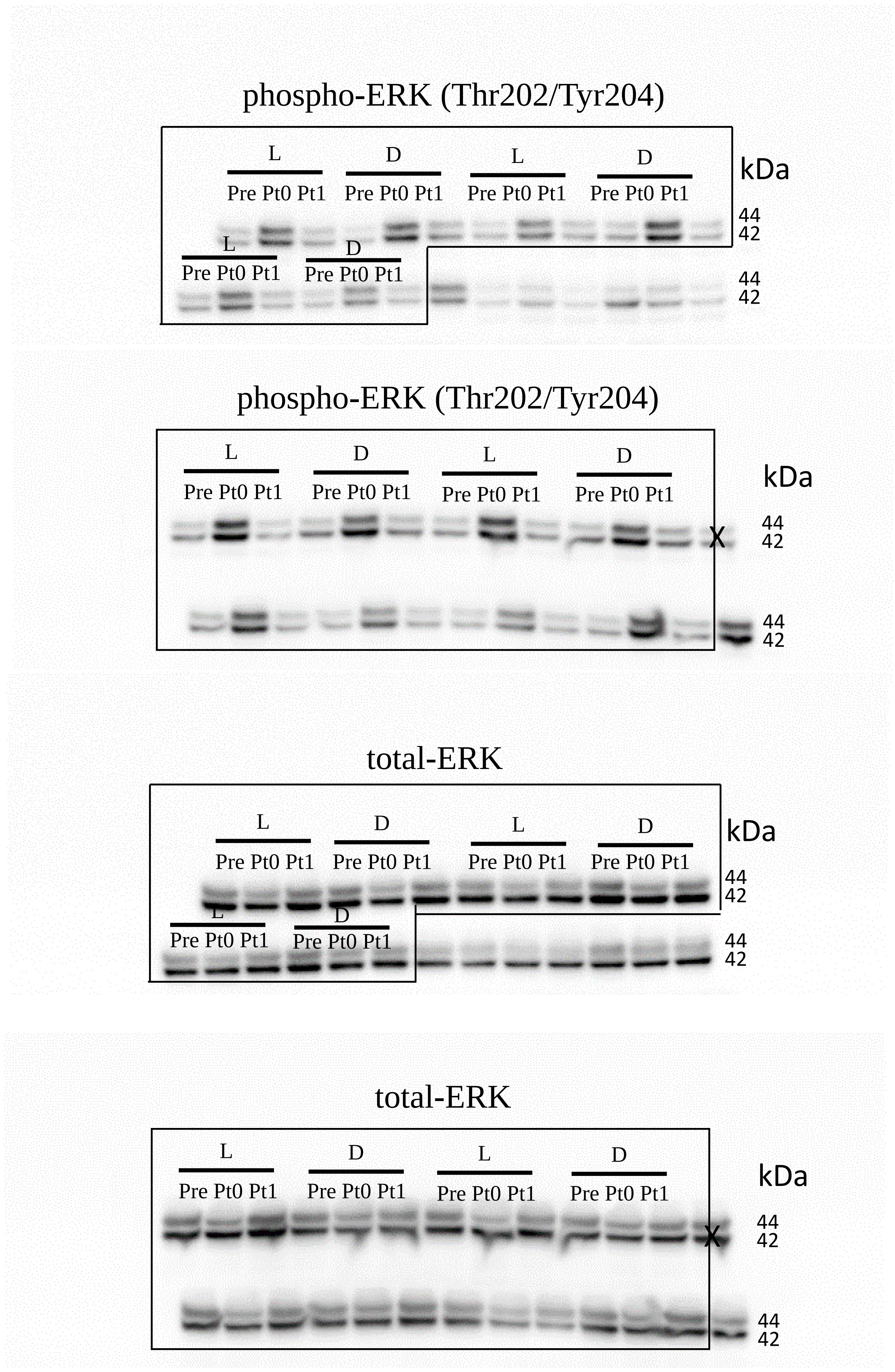
phospho-ERK (Thr202/Tyr204)
L D L D
Pre Pt0 Pt1 Pre Pt0 Pt1 Pre Pt0 Pt1 Pre Pt0 Pt1
kDa
44
42
L D
Pre Pt0 Pt1 Pre Pt0 Pt1
44
42
phospho-ERK (Thr202/Tyr204)
L D L D
Pre Pt0 Pt1 Pre Pt0 Pt1 Pre Pt0 Pt1 Pre Pt0 Pt1
kDa
X
44
42
44
42
total-ERK
L D L D
Pre Pt0 Pt1 Pre Pt0 Pt1 Pre Pt0 Pt1 Pre Pt0 Pt1
kDa
44
42
L D
Pre Pt0 Pt1 Pre Pt0 Pt1
44
42
total-ERK
L D L D
Pre Pt0 Pt1 Pre Pt0 Pt1 Pre Pt0 Pt1 Pre Pt0 Pt1
kDa
X
44
42
44
42

Fig 3d
