# Supplementary material for: Circadian rhythms modulate the effect of eccentric exercise on rat soleus muscles
Source: PLoS One. 2022 Feb 25;17(2):e0264171. doi: 10.1371/journal.pone.0264171 (PMC8880858; doi:10.1371/journal.pone.0264171)
Supplement: Attachment — Submitted filename: PONE_Responses_to_Reviewer20210701-5.docx [file pone.0264171.s003.docx]

PONE-D-21-07409

Circadian rhythms modulate the effect of eccentric exercise on rat skeletal muscles via the mTOR signaling pathway

*PLOS ONE*

We wish to express our appreciation to the reviewers for their insightful comments on our manuscript. Their comments have helped us significantly improve our paper. We have attempted to address all their concerns. Our point-by-point responses to the comments are given below.

**Responses to Reviewer #1**

***Comment:*** This study aimed to investigate the effect of circadian rhythms on exercise-mediated muscle anabolism. The authors concluded that the degree of eccentric exercise-mediated muscle hypertrophy in soleus muscle is time-of-day dependent and circadian rhythms of corticosterone and mTOR/p70S6K signaling affect this adaptation. Although this is an interesting project that will bring important insight to our field, there are many concerns.

***Comment 1:*** L46-48: The authors do not follow the current dogma that mTORC1/p70S6K is not a major signaling pathway in the regulation of contraction-induced protein synthesis. Also, ref. 10 does not report the correlation between signaling and muscle hypertrophy. The authors should revise this point and further add the reference that ERK signaling contributes to the contraction-induced protein synthesis.

***Response 1:*** Thank you for your suggestion. There are many signaling pathways that promote muscle growth, such as Insulin/IGF1-AKT-mTOR, TGFβ/myostatin/activin/BMP, β-adrenergic signaling, FGF/zinc ions, and desmosomes/Zinc ions (Sartori et al., 2021). However, until recently, the mTORC1/p70S6K (Kotani et al., 2021; Ashida et al., 2018; Ribeiro et al., 2017) and ERK (Williamson, et al., 2003; Takegaki et al., 2019) signaling have still been used as an indicator for the synthesis of muscle proteins. Therefore, we believe that both mTORC1/p70S6K and ERK signaling are important. We will investigate the other contraction-induced protein synthesis pathways in the future.

We have included the abovementioned information in the revised text and cited the relevant references (p. 3, lines 47–48; p. 16–17, lines 273–280).

References:

Sartori, R., Romanello, V., & Sandri, M. (2021). Mechanisms of muscle atrophy and hypertrophy: Implications in health and disease. Nature Communications, 12(1), 1-12.

Kotani, T., Takegaki, J., Tamura, Y., Kouzaki, K., Nakazato, K., & Ishii, N. (2021). The effect of repeated bouts of electrical stimulation-induced muscle contractions on proteolytic signaling in rat skeletal muscle. Physiological Reports, 9(9), e14842.

Ashida, Y., Himori, K., Tatebayashi, D., Yamada, R., Ogasawara, R., & Yamada, T. (2018). Effects of contraction mode and stimulation frequency on electrical stimulation-induced skeletal muscle hypertrophy. Journal of Applied Physiology, 124(2), 341-348.

Ribeiro, M. B. T., Guzzoni, V., Hord, J. M., Lopes, G. N., de Cássia Marqueti, R., de Andrade, R. V., ... & Durigan, J. L. Q. (2017). Resistance training regulates gene expression of molecules associated with intramyocellular lipids, glucose signaling and fiber size in old rats. Scientific Reports, 7(1), 1-13.

Williamson, D., Gallagher, P., Harber, M., Hollon, C., & Trappe, S. (2003). Mitogen-activated protein kinase (MAPK) pathway activation: effects of age and acute exercise on human skeletal muscle. The Journal of Physiology, 547(3), 977-987.

Takegaki, J., Sase, K., & Fujita, S. (2019). Repeated bouts of resistance exercise attenuate mitogen-activated protein-kinase signal responses in rat skeletal muscle. Biochemical and Biophysical Research Communications, 520(1), 73-78.

***Comment 2:*** L68: Why did the authors only investigate soleus muscle? This point significantly reduces the value of this paper. Generally, downhill running affects not only soleus muscle but also gastrocnemius (and plantalis) muscle and therefore the authors should add the data of gastrocnemius at least the results of chronic training.

***Response 2:*** Thank you for your suggestion. We have presented the observations made in the gastrocnemius and plantalis muscles in Table 1 (p. 11–12, lines 192–196). We have indicated that only soleus, for which interaction was found, was used in the analysis because there was no interaction or other effect of downhill training on gastrocnemius or plantalis.

***Comment 3:*** L84-87: Was exercise intensity constant?

***Response 3:*** Yes, it was constant. The program for each training session was 16 m/min (incline: -16°) and 90 minutes of exercise time.

***Comment 4:*** L95-96: Ref. 4 (your previous study) reported that mTOR signaling is high at ZT6 as compared with at ZT18 in plantaris but not soleus.

***Response 4:*** In the SOL, there was no significant difference, but there was a 49.5% difference observed between the two points near the highest and lowest values; thus, even if there was no difference at rest (or even if the difference was not large), the effects of exercise could still be different.

***Comment 5:*** L100: Why did the authors use different exercise protocols between acute and chronic exercise?

***Response 5:*** Although the increase and duration of the signal may be different when the time is shortened, the time is shortened because it helps obtain an understanding of the signaling pathways involved. We decided that it is not necessary to use the same protocol in order to see the response reflected one time because each response will be different during the adaptation process.

***Comment 6:*** L123-: Is 99% glycerol correct? What does “samples containing total protein” mean?

***Response 6:*** Thank you for pointing this out. The final concentration was 30% glycerol, 5% 2-mercaptoethanol, 2.3% SDS, 62.5 mm Tris–HCl pH 6.8, and bromphenol blue. We have corrected the relevant text in the revised manuscript (p. 8, lines 128–129).

“Samples containing total protein” are samples of mixtures of protein extracts and sample buffer.

***Comment 7:*** L156-162: Why is there no difference in body weight between the control and exercise training groups when the control rats eat a lot and do not exercise? Need discussion and add fat mass data if possible.

***Response 7:*** We agree with the reviewer’s contention, and acknowledge that the suggestion made by the reviewer is valuable. We have added the fat mass data (revised Fig. 1) and have discussed this issue in the Discussion section of the revised manuscript (p. 15, lines 248–260).

We noticed that the information on food intake in the original manuscript was incorrect. We apologize for this oversight and have corrected it in the revised manuscript. The food intake in the control group was almost the same as that in the training group; however, rats in the control group in this study had more body fat (epididymal white adipose tissue) than those in the training group.

***Comment 8:*** L187-191: mTOR S2448 phosphorylation does not necessarily associate mTORC1 activity. The authors can evaluate mTORC1 activity more appropriately to measure both p70S6K and 4E-BP1 phosphorylation.

***Response 8:*** We agree with the valuable comment made by the reviewer. We added 4E-BP1 data and modified the relevant text in the revised manuscript (revised Fig. 3 and p. 17, lines 281–290).

***Comment 9:*** L202-204: The authors report only a %change in serum corticosterone concentration. Please add absolute values at pre- and post-exercise.

***Response 9:*** Thank you for your suggestion. We have added the serum corticosterone concentration data (revised Fig. 4 and p. 14, lines 225–239).

***Comment 10:*** L217-226: Resistance exercise increases mTORC1 signaling for more than 24h after exercise while endurance exercise activates mTORC1 signaling only immediately after exercise. The results of this study are in accordance with the signaling responses of endurance exercise rather than resistance exercise, suggesting that factors other than mTORC1 signaling associate downhill running-induced muscle hypertrophy.

***Response 10:*** Thank you for your keen analysis. The signaling pattern described in the comment is certainly true for humans, but that in animals can also be degraded in a relatively short time in response to hypertrophy-inducing movements, such as electrical stimulation (Bolster et al., 2003). However, we believe that the timing of the exercise is also important. In this study, we assessed the effects of a bout of exercise on Wistar rats to check whether exercising at different times (ZT6 or ZT18) had different effects on mTOR/p70S6K signaling.

References:

Bolster, D. R., Kubica, N., Crozier, S. J., Williamson, D. L., Farrell, P. A., Kimball, S. R., & Jefferson, L. S. (2003). Immediate response of mammalian target of rapamycin (mTOR)‐mediated signalling following acute resistance exercise in rat skeletal muscle. The Journal of physiology, 553(1), 213-220.

***Comment 11:*** L228: Ref. 4 does not report the role of ERK signaling in the mechanisms of muscle hypertrophy.

***Response 11:*** Ref. 4 was not cited on line 228. In Ref. 4, we described that the physiological significance of circadian variation in ERK phosphorylation in the cardiac and plantaris muscles remains unclear. Based on the results of transient exercise at different times in this study, there was no difference in ERK signaling, suggesting that this signaling is less likely as a pathway for exercise-induced muscle hypertrophy in the light period. Nevertheless, the results of ERK signaling in this study can prove that the intensity of exercise performed at different times is the same.

We have modified the relevant text in the revised manuscript because our explanation may be inadequate (p. 16–17, lines 277–280).

***Comment 12:*** L236-253: Regarding corticosterone, the authors state that serum corticosterone may be associated mTOR/p70S6K signaling, as it has a role in muscle protein breakdown. Please discuss more specifically with the references. How muscle protein breakdown affects mTOR signaling?

Serum corticosterone response may affect the mTOR signaling response but how about the resting condition? Not resting (absolute value) but only responses affect mTOR signaling?

It is interesting and important to measure REDD1 to know the mechanisms of reduced mTORC1 responses.

***Response 12:***

Thank you for your suggestion. Considering muscle protein degradation affects mTOR signaling, we have added a discussion to the revised manuscript (p. 17–18, lines 297–306).

The serum corticosterone concentration in the light period (ZT6) group was generally higher than that in the dark period (ZT18) group, possibly due to the interconnection between circadian rhythm and corticosterone concentration that peaked around ZT11-13. In contrast, the corticosterone peak in the dark period (ZT18) group was past and the concentration had lowered. The serum corticosterone levels in LTR and DLR groups immediately after exercise were not significantly different, but the rate of increase in corticosterone concentration immediately after exercise was greater in the dark-phase group than in the light-phase group (revised Fig. 4). These observations tentatively suggest that corticosterone has a slightly antagonistic impact on mTOR/p70S6K signaling during the light phase, explaining the increase in exercise-induced p70S6K phosphorylation and subsequent muscle hypertrophy during that time of day (p. 18–19, lines 310–317). However, the underlying mechanisms are unknown, and would be investigated in the future.

We tried to analyze the expression of REDD1 (35 kDa) using antibodies against it (10638-1-AP) purchased from Proteintech Group, Inc., but could not detect clear bands (as shown in the figure below).

***Comment 13:*** L260-265: Obviously, exercise type and fiber type affect the signaling responses. However, the authors suggested that different adaptation to the training is led by serum corticosterone (systemic factor). Therefore, the authors should discuss not only signaling but also systemic factors.

***Response 13:***

Thank you for your suggestion. We have added the following discussion to the revised manuscript (p. 19, lines 334–338):

Moreover, hormones as indicators of systemic factors also affect muscle mass; for example, differences in hormone levels in the body (testosterone:corticosterone ratio) or differences in hormone levels caused by exercise. Therefore, the effect of systemic factors on muscle mass cannot be ignored (Crowley & Matt, 1996; Guzzoni, 2019).

***Comment 14:*** L268: Which paper shows that the timing of feeding is important to induce muscle hypertrophy?

***Response 14:*** Thank you for this query. We have cited the following reference (p. 20, line 341):

Phillips, S. M., Tipton, K. D., Aarsland, A., Wolf, S. E., & Wolfe, R. R. (1997). Mixed muscle protein synthesis and breakdown after resistance exercise in humans. American Journal of Physiology, 273(1), E99-E107.

***Comment 15:*** L269: Ref. 30 is a review paper. So please add the original article(s).

***Response 15:*** Thank you for your suggestion. We have cited the following original article (p. 20, line 344):

Burd, N. A., West, D. W., Moore, D. R., Atherton, P. J., Staples, A. W., Prior, T., ... & Phillips, S. M. (2011). Enhanced amino acid sensitivity of myofibrillar protein synthesis persists for up to 24 h after resistance exercise in young men. The Journal of Nutrition, 141(4), 568-573.

**Responses to Reviewer #2**

***Comment 1:*** Please if it is possible to show the circadian clock proteins or the gene expression, since exercise could have modified your circadian clock (i.e Bmal1 / CLOCK). If you can't do this experiment please discuss.

***Response 1:*** Thank you for your suggestion. Unfortunately, we could not measure the gene expression in this study because of limitation of the samples. Therefore, we have added relevant discussion about it in the revised manuscript. (p. 21, lines 367–373).

The clock gene has been reported to be involved in the structure and function of skeletal muscle (Andrews et al., 2010). It has also been demonstrated that exercise affects the circadian clock (Bmal1/CLOCK) of skeletal muscle (Wolff et al., 2012). Based on these studies, it may be possible to influence muscles from exercise via the clock genes. As per recent studies, some pathways are likely to influence Per1 by glucocorticoids via REDD1 (Saracino et al., 2019). Another pathway possibly affects BMAL1 through the activation of mTORC1 (Dadon-Freiberg et al., 2021). However, further research is needed to confirm whether these pathways affect the muscle mass.

***Comment 2:*** Please detail the food intake in a better way (how much they ate in the night phase, how much they ate in the light phase, adjust the food intake to the weight of the animal ...) and discuss, this is a very important point of the study. I suggest making a table with date of food intake.

***Response 2:*** Thank you for your comments. We measured food intake, without food and water restrictions, after a single training session of 3 days. The weekly food intake for each group is shown in the revised figure 1. Unfortunately, we are unable to present the detailed feed intake. To avoid disrupting the biological rhythms and to minimize non-training stresses (entering and exiting the animal house, turning lights on and off, etc.) in the experimental animals, we took only a limited number of measurements. It has also been previously reported that exposure to light and food restriction alters the periodicity of endocrine, body temperature, and activity in experimental animals (Mohawk et al., 2007; Wideman et al., 2009; Depres-Brummer et al., 1995; Krieger et al., 1974). In addition, it is reported that factors like exercise and food restriction were stressful for experimental animals and affected the secretion of corticosterone (Fediuc, 2006; Heiderstadt, 2000). However, although we do not know exactly when the animals ate, there was no notable difference observed in food intake.

***Comment 3:*** Please take better advantage of the H&E, measure the number of membrane nuclei and central nuclei, remembering that eccentric exercise generates high muscle damage.

***Response 3:*** We are thankful for the valuable suggestion. We have added the data on the central nucleus as an indicator of regenerating muscle (revised Fig. 2) and have discussed this issue in the Discussion section of the revised manuscript (p. 21, lines 358–366).

Upon the onset of muscle damage, satellite cells are activated; they proliferate and differentiate, eventually fusing with other satellite cells to form myofibers with central nuclei (Sasaki et al., 2007; Yu et al., 2017). Therefore, the central nuclei (shown as the ratio of the centronucleated fiber) are used as an indicator of regenerating muscle. It was previously reported that the central nucleus of the soleus muscle of rats was clearly observed after 5 days of a bout of downhill running (Yu et al., 2017). However, in this study, there was no difference in the central nucleus in any of the groups because we used the same training intensity and training time for 8 weeks. Therefore, the muscles would be accustomed to the training stimulus, and damage would be less likely to occur.

***Comment 4:*** Since you have individual corticosterone values (changes) and you believe that it is responsible for your cellular results, please make correlations between changes in corticosterone and changes in protein signaling, CSA and strength level.

***Response 4:*** Thank you for your suggestion. We acknowledge that the analyses suggested by you would provide great insights. However, we could not determine the correlations between the variables because these data were obtained from different rats (i.e., they are not matching data).
