# Supplementary material for: Circadian rhythms modulate the effect of eccentric exercise on rat soleus muscles
Source: PLoS One. 2022 Feb 25;17(2):e0264171. doi: 10.1371/journal.pone.0264171 (PMC8880858; doi:10.1371/journal.pone.0264171)
Supplement: Attachment — Submitted filename: PONE_Responses_to_Reviewer20220116-1.docx [file pone.0264171.s004.docx]

We appreciate the insightful comments from the reviewers. Their comments have helped us improve our paper. We have attempted to address all their concerns. Our point-by-point responses to the comments are given below.

Reviewer #1:

Comment 1: L46-48: The authors do not follow the current dogma that mTORC1/p70S6K is not a major signaling pathway in the regulation of contraction-induced protein synthesis. Also, ref. 10 does not report the correlation between signaling and muscle hypertrophy. The authors should revise this point and further add the reference that ERK signaling contributes to the contraction-induced protein synthesis.
Response 1: Thank you for your suggestion. There are many signaling pathways that promote muscle growth, such as Insulin/IGF1-AKT-mTOR, TGFβ/myostatin/activin/BMP, β-adrenergic signaling, FGF/zinc ions, and desmosomes/Zinc ions (Sartori et al., 2021). However, until recently, the mTORC1/p70S6K (Kotani et al., 2021; Ashida et al., 2018; Ribeiro et al., 2017) and ERK (Williamson, et al., 2003; Takegaki et al., 2019) signaling have still been used as an indicator for the synthesis of muscle proteins. Therefore, we believe that both mTORC1/p70S6K and ERK signaling are important. We will investigate the other contraction-induced protein synthesis pathways in the future.
We have included the abovementioned information in the revised text and cited the relevant references (p. 3, lines 47–48; p. 16–17, lines 273–280).

***Comment 1-1***: It’s not scientific. They might use mTORC1 and ERK as an indicator but mTORC1/p70S6K is not a major signaling pathway in the regulation of contraction-induced protein synthesis (PMID: 26227152, 30509128. Please note that PMID: 30509128 reported that while the contraction-induced protein synthesis is mTORC1-independent, muscle hypertrophy is mTORC1-dependent). Also, there is no evidence that ERK regulates contraction-induced muscle protein synthesis, and rather that is denied (PMID: 23077579). Therefore, of course, the authors can use mTORC1 as an indicator but state and discuss carefully based on the fact because the authors did not measure muscle protein synthesis.

***Response 1-1:***

We thank the reviewer for this suggestion. We acknowledge that in the study by You et al. (2019), protein synthesis by mechanical loading is mTORC1-independent, but muscle hypertrophy is mTORC1-dependent. However, some previous studies have shown that exercise-induced protein synthesis involves mTORC1. A study in mice by Ogasawara et al. (2018) reported that resistance exercise (transcutaneous electrical stimulation to induce muscle contraction) increases muscle protein synthesis via rapamycin-sensitive mTORC1 and rapamycin-insensitive mTORC1 or mTORC2. The difference in these results might be due to the differences in the pathways of muscle protein synthesis induced by different stimuli leading to muscle hypertrophy like electrical stimulation or myotenectomy. In this study, we did not measure muscle protein synthesis; therefore, the relationship between mTORC1 and muscle protein synthesis by downhill running is currently unknown. Following the reviewer's advice, we have described this in the Discussion section.

We have added the following discussion to the revised manuscript (p. 19–20, lines 327–339):

“Previous studies have shown a relationship between mTOR/p70S6K and muscle protein synthesis. A mouse study by Ogasawara et al. (2018) reported that the promotion of muscle protein synthesis by resistance exercise (transcutaneous electrical stimulation to induce muscle contraction) is regulated by mTOR (both rapamycin-sensitive mTORC1 and rapamycin-insensitive mTORC1 or mTORC2). However, a recent study reported that exercise-induced protein synthesis is independent of mTOR/p70S6K. You et al. (2019) reported that protein synthesis by mechanical loading is mTORC1-independent in the case of myotenectomy-induced muscle hypertrophy in mice. Different experimental methods of inducing muscle hypertrophy-like electrical stimulation or myotenectomy-induced muscle hypertrophy may also result in different pathways of muscle protein synthesis. Therefore, the relationship between mTOR/p70S6K and muscle protein synthesis remains unclear. In this study also we have not elucidated the relationship between mTOR/p70S6K as we have not measured muscle protein synthesis.”

References:

Ogasawara, R., & Suginohara, T. (2018). Rapamycin‐insensitive mechanistic target of rapamycin regulates basal and resistance exercise‐induced muscle protein synthesis. The FASEB Journal, 32(11), 5824-5834.

You, J. S., McNally, R. M., Jacobs, B. L., Privett, R. E., Gundermann, D. M., Lin, K. H., ... & Hornberger, T. A. (2019). The role of raptor in the mechanical load‐induced regulation of mTOR signaling, protein synthesis, and skeletal muscle hypertrophy. The FASEB Journal, 33(3), 4021-4034.

Comment 2: L68: Why did the authors only investigate soleus muscle? This point significantly reduces the value of this paper. Generally, downhill running affects not only soleus muscle but also gastrocnemius (and plantalis) muscle and therefore the authors should add the data of gastrocnemius at least the results of chronic training.
Response 2: Thank you for your suggestion. We have presented the observations made in the gastrocnemius and plantalis muscles in Table 1 (p. 11–12, lines 192–196). We have indicated that only soleus, for which interaction was found, was used in the analysis because there was no interaction or other effect of downhill training on gastrocnemius or plantalis.

***Comment 2-1:*** Please discuss why exercise in this study induced muscle hypertrophy only in soleus muscle despite eccentric exercise predominantly activates fast-twitch fibers. Based on this result, the author should change the title from “rat skeletal muscle” to “rat soleus muscle”

***Response 2-1:***

We thank the reviewer for this suggestion. We have added the following description to the revised manuscript (p. 21–22, lines 368–377) and changed the title to ‘rat soleus muscle’.

The previous study on rats has shown that most of the fiber components of the soleus muscle are mobilized during downhill running (incline: -16°) (Smith et al., 1997). In addition, when low-intensity exercise (10-20 m/min) is performed with downhill running (treadmill grade 15% decline), fast-twitch muscles might be involved to a much lesser extent in the motor unit recruitment (Dudley et al., 1982). Therefore, the cause of the induced muscle hypertrophy only in the soleus muscle could be determined as only the low-intensity exercise was involved in this study. It is thought that low-intensity exercise may not reflect the effects of circadian rhythm on fast-twitch muscles as the fast-twitch muscles are scarcely stimulated by low-intensity exercise. In the future, employment of high-intensity exercise will be required to prove this.”

References:

Smith, H. K., Plyley, M. J., Rodgers, C. D., & McKee, N. H. (1997). Skeletal muscle damage in the rat hindlimb following single or repeated daily bouts of downhill exercise. International journal of sports medicine, 18(02), 94-100.

Dudley, G. A., Abraham, W. M., & Terjung, R. L. (1982). Influence of exercise intensity and duration on biochemical adaptations in skeletal muscle. Journal of applied physiology, 53(4), 844-850.

Comment 4: L95-96: Ref. 4 (your previous study) reported that mTOR signaling is high at ZT6 as compared with at ZT18 in plantaris but not soleus.
Response 4: In the SOL, there was no significant difference, but there was a 49.5% difference observed between the two points near the highest and lowest values; thus, even if there was no difference at rest (or even if the difference was not large), the effects of exercise could still be different.

***Comment 4-1:*** Even if there was a relatively large difference, if there was no significant difference, the authors should not be stated that there is a difference.

***Response 4-1:***

We apologize for the inadequate explanation in our previous reply. The statistically significant difference described in our previous study (Reference no. 4) was calculated using a statistical method of the modified cosinor analysis to determine the existence of a circadian rhythm. This is different from the tests of differences between means that are usually used (e.g., t-test). Therefore, we think that the statement in our previous publication, "Circadian rhythms of signal transducers were observed in both cardiac (mTOR, p70S6K, and ERK) and plantaris (p70S6K and ERK) muscles (p < 0.05), but not in the soleus muscle." is reasonable. In addition, in the cited study (Reference no. 4), we do not claim that the mTOR signaling is high at ZT6 as compared with at ZT18 in plantaris but not soleus.

Comment 10: L217-226: Resistance exercise increases mTORC1 signaling for more than 24h after exercise while endurance exercise activates mTORC1 signaling only immediately after exercise. The results of this study are in accordance with the signaling responses of endurance exercise rather than resistance exercise, suggesting that factors other than mTORC1 signaling associate downhill running-induced muscle hypertrophy.
Response 10: Thank you for your keen analysis. The signaling pattern described in the comment is certainly true for humans, but that in animals can also be degraded in a relatively short time in response to hypertrophy-inducing movements, such as electrical stimulation (Bolster et al., 2003). However, we believe that the timing of the exercise is also important. In this study, we assessed the effects of a bout of exercise on Wistar rats to check whether exercising at different times (ZT6 or ZT18) had different effects on mTOR/p70S6K signaling.

***Comment 10-1:*** Bolster et al 2003 used a squat model. They used ES to impose a jump but not to directly stimulate muscle activation. This squat model does not induce muscle hypertrophy and is currently not used as a resistance training model.

***Response 10-1:***

We apologize for the lack of explanation. As pointed out by the reviewer, the study by Bolster et al. (2003) is an experiment in which rats were made to jump using electrical stimulation. In addition, a previous study by Nader et al. (2001) used low-frequency electrical stimulation to increase mTORC1 signaling (p70S6K), which returned to baseline 6 hours after stimulation. We believe that different exercise intensities cause distinct changes in signaling.

Reference:

Nader, G. A., & Esser, K. A. (2001). Intracellular signaling specificity in skeletal muscle in response to different modes of exercise. Journal of applied physiology, 90(5), 1936-1942.

Comment 12: L236-253: Regarding corticosterone, the authors state that serum corticosterone may be associated mTOR/p70S6K signaling, as it has a role in muscle protein breakdown. Please discuss more specifically with the references. How muscle protein breakdown affects mTOR signaling?
Serum corticosterone response may affect the mTOR signaling response but how about the resting condition? Not resting (absolute value) but only responses affect mTOR signaling?
It is interesting and important to measure REDD1 to know the mechanisms of reduced mTORC1 responses.
Response 12:
Thank you for your suggestion. Considering muscle protein degradation affects mTOR signaling, we have added a discussion to the revised manuscript (p. 17–18, lines 297–306).

***Comment 12-1:*** The main point was that how muscle protein breakdown affects mTOR. It is well known that corticosterone regulates protein breakdown and mTOR, respectively, but it is unclear how protein breakdown affects mTOR. Please delete the following sentence: “as it has a well-known role in the breakdown of muscle proteins.

***Response 12-1:***

As per the reviewer’s suggestion, we have now deleted the text (p. 17, lines 291–293).

Comment 14: L268: Which paper shows that the timing of feeding is important to induce muscle hypertrophy?
Response 14: Thank you for this query. We have cited the following reference (p. 20, line 341):
Phillips, S. M., Tipton, K. D., Aarsland, A., Wolf, S. E., & Wolfe, R. R. (1997). Mixed muscle protein synthesis and breakdown after resistance exercise in humans. American Journal of Physiology, 273(1), E99-E107.

***Comment 14-1:*** They did not investigate the timing of feeding. Also, they did not measure muscle size.

***Response 14-1:***

Thank you for pointing this out. We have now replaced the references. We have also revised the text to avoid misunderstanding in the revised manuscript as follows (p. 20, lines 350–352):

“Although previous studies in human subjects have demonstrated that the timing of feeding post-exercise (1-3 hours after training) plays an important role in promoting protein synthesis (Rasmussen et al., 2000),…..”

Reference:

Rasmussen, B. B., Tipton, K. D., Miller, S. L., Wolf, S. E., & Wolfe, R. R. (2000). An oral essential amino acid-carbohydrate supplement enhances muscle protein anabolism after resistance exercise. Journal of applied physiology, 88(2), 386-392.

***Finally***, please consider changing the title from “via the mTOR…” to “possibly via the mTOR…” or delete “via the mTOR…” because the authors have not proven causation.

***Response:***

As per the reviewer's suggestion, we have now changed the title to “Circadian rhythms modulate the effect of eccentric exercise on rat soleus muscles.”

Reviewer #2: (No Response)
